# Surgical treatment for late-appearing adrenal metastasis from gastric cancer: report of two cases

**DOI:** 10.1186/1477-7819-12-116

**Published:** 2014-04-24

**Authors:** Dong Jin Kim, Jun Hyun Lee, Wook Kim

**Affiliations:** 1 Department of Surgery, Yeouido St Mary’s Hospital, The Catholic University of Korea, 62 Yeouido-dong, Yeongdeungpo-gu, Seoul 150-713, Korea; 2 Department of Surgery, Bucheon St Mary’s Hospital, College of Medicine, The Catholic University of Korea, 62 Yeouido-dong, Yeongdeungpo-gu, Seoul 150-713, Korea

**Keywords:** Adrenal gland neoplasm, Gastrectomy, Stomach neoplasm

## Abstract

Adrenal metastasis following gastrectomy for gastric cancer is often encountered as part of advanced systemic dissemination, which is usually unresectable. Thus, there are very few published case reports describing metastasectomy for adrenal metastasis from gastric cancer. Herein we present our experience in treating two patients diagnosed and treated for adrenal metastasis 6 years following initial surgery for advanced gastric cancer (pT2bN1M0 and pT2bN0M0, respectively, according to the classification system set forth in the sixth edition of *The TNM Classification of Malignant Tumours* by the International Union against Cancer). They underwent successful *en bloc* R0 resections, followed by systemic chemotherapy with close postoperative follow-up for another recurrence, and have remained alive without recurrence for 1 year. These results suggest that active surgical treatment for resectable metastatic gastric cancer in the adrenal glands has an important role in prolonging survival in selected patients.

## Background

Common recurrence patterns following curative resection for advanced gastric cancer (AGC) are peritoneal dissemination and hematogenous metastasis. The liver is the main focus among extraperitoneal metastases, and the lung is the secondary focus [1,2]. Although some autopsy studies have shown that 16% to 18% of patients with gastric cancer developed adrenal metastases [3,4], most occurred in connection with multiple synchronous metastases to other sites [5]. Herein we report the cases of two patients with late-onset single adrenal metastases 6 years after gastrectomy for AGC who underwent successful curative resection.

## Case presentations

### Case 1

A 51-year-old man visited our hospital for a regular postoperative follow-up examination for gastric cancer in April 2012. He had undergone a total gastrectomy for AGC on the cardia in September 2006 (pT2bN1M0, stage II, according to the classification system set forth in the sixth edition of *The TNM Classification of Malignant Tumours* by the International Union against Cancer (UICC) [6]). At that time, the histological examination revealed a poorly differentiated tubular adenocarcinoma that had infiltrated the subserosal layer. Thirty-five lymph nodes were retrieved, and one was metastatic. After an R0 resection, the patient received six cycles of cisplatin and 5-fluorouracil (5-FU) combination chemotherapy until March 2007. He was followed up regularly with tumor markers, abdominal computed tomography (CT) scans and positron emission tomography (PET)-CT every 6 months for 2 years and then annually thereafter.

**Figure 1:**
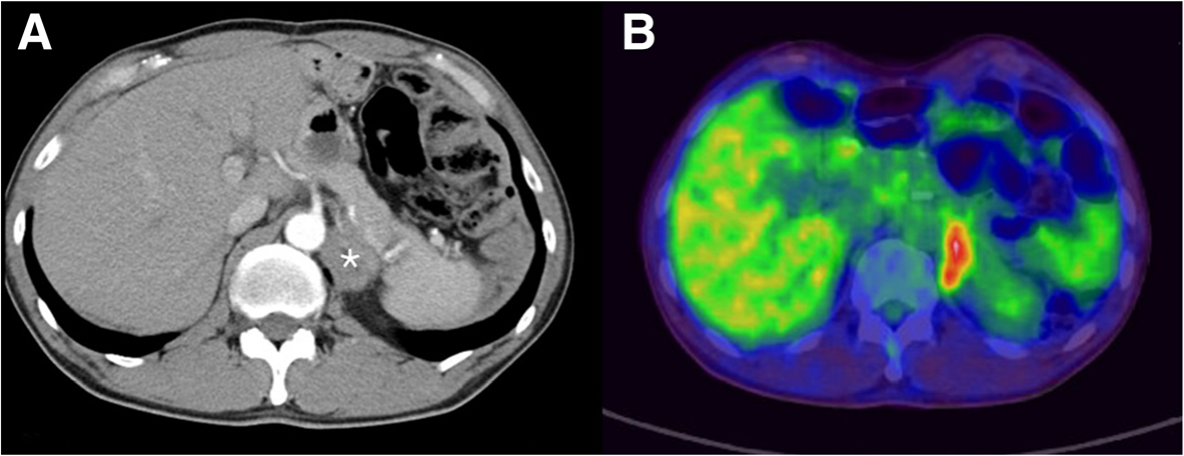
**Follow-up computed tomography and positron emission tomography–computed tomography scans of patient 1 obtained in September 2011 show a 3-cm left adrenal mass (asterisk). (A)** Computed tomography shows accidentally detected left adreanal mass. **(B)** PET-CT shows significant FDG uptake area correlated with enlarged left adenal mass.

**Figure 2:**
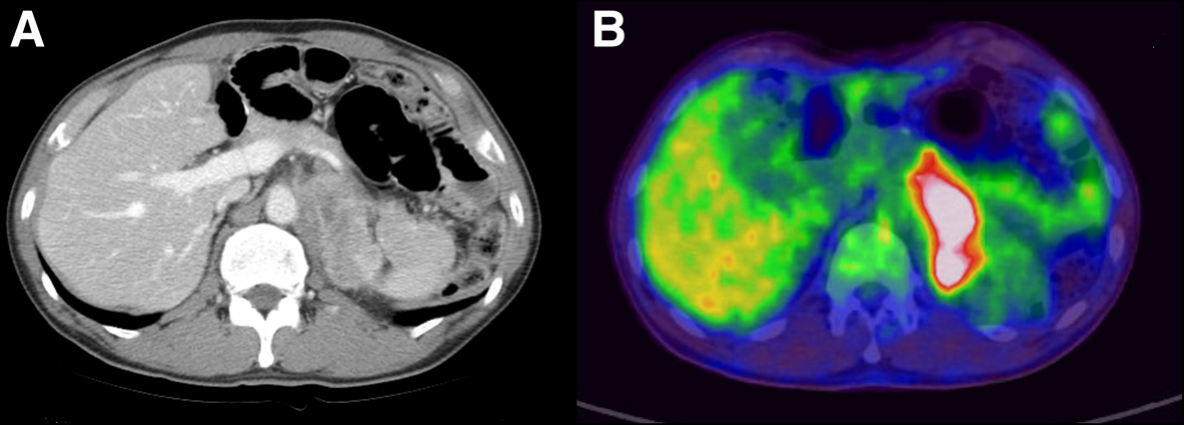
**Follow-up computed tomography and positron emission tomography–computed tomography scans of patient 1 obtained in March 2012 show an increase in the size of the left adrenal mass. (A)** Computed tomography image shows left adrenal mass interval increased. **(B)** PET-CT shows interval increased FDG hot uptake area.

**Figure 3:**
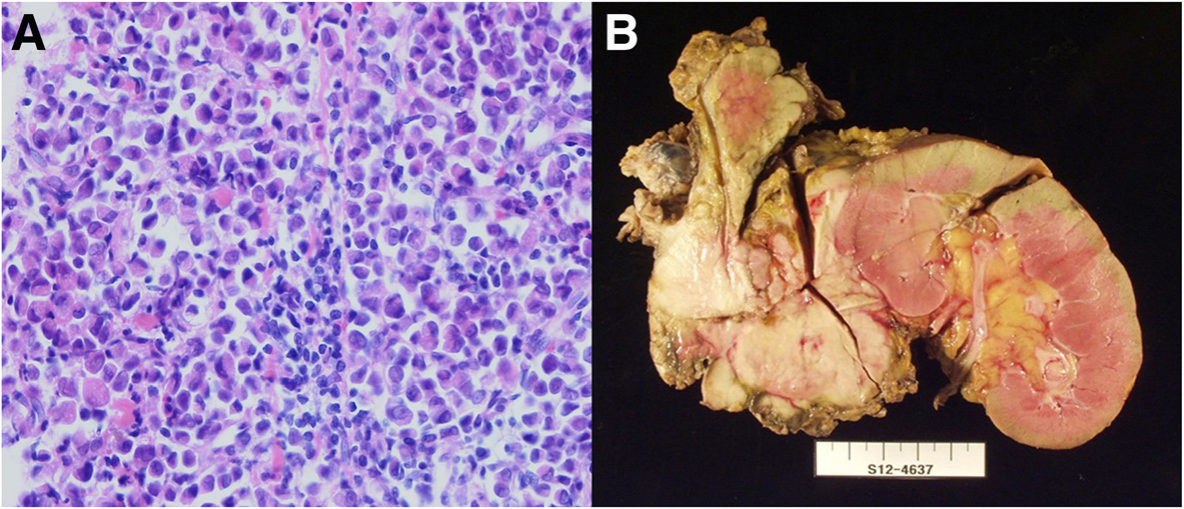
**Histological tissue specimen and photograph of tumor showing poorly differentiated adenocarcinoma in case 1. (A)** We evaluated this computed tomography–guided biopsy specimen histologically (hematoxylin and eosin stain; original magnification, ×400). **(B)** Photograph of the resected specimen from patient 1 shows a 6-cm adrenal mass invading the pancreas and the renal parenchyma.

The patient had no symptoms or abnormal findings suggesting recurrence until 5 years postoperatively, in September 2011. An abdominal CT scan showed an isolated left adrenal mass, 3 cm in size, with inhomogeneous enhancement, which was suspected to be a relapse of the disease or an adrenal incidentaloma (Figure 1A). He was recommended for a follow-up examination because tumor markers were within normal limits and there was only minimal fluorodeoxyglucose (FDG) uptake visible on the PET scan (Figure 1B). Six months later a CT scan showed that the preexisting adrenal mass had increased in size (up to 6 cm), and PET-CT scans showed a more definite hypermetabolic focus (Figure 2). A CT-guided biopsy of the tumor was performed for histological confirmation, which revealed a poorly differentiated adenocarcinoma compatible with metastasis from gastric cancer (Figure 3A). Following sufficient exploration for surgical resection and other alternative treatments, surgical resection was planned with the patient’s informed consent.

In the patient’s second operation, 6 years after initial operation, the left adrenal tumor was found to have extended to the adjacent organs: to the distal pancreas anteriorly, to the renal capsule inferiorly and to the diaphragm superiorly. An *en bloc* resection, including the left adrenal gland, kidney, a portion of the diaphragm, distal pancreas and spleen, was performed (Figure 3B). Histopathological examination revealed a poorly differentiated metastatic adenocarcinoma invading the renal capsule and distal pancreas. Other than the main tumor, we detected three metastatic lymph nodes among a total of eight retrieved lymph nodes in the paraaortic region. The patient’s postoperative course was uneventful, and he was discharged on postoperative day 13. He subsequently received eight cycles of second-line chemotherapy with 120 mg/day S-1 (TS-1; Taiho Pharmaceutical, Tokyo, Japan). During the 13-month follow-up period, there was no evidence of cancer recurrence based on CT findings and tumor markers.

### Case 2

**Figure 4:**
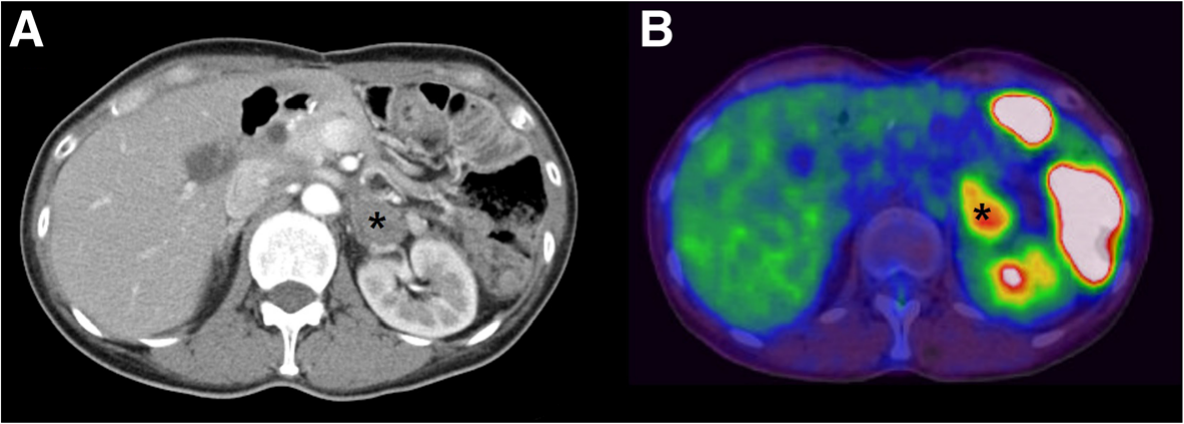
**Computed tomography and positron emission tomography–computed tomography scans of patient 2 obtained in March 2012 show a 3-cm hypermetabolic left adrenal mass (asterisk). (A)** Computed tomography shows well circumscribed left adrenal mass. **(B)** PET-CT shows FDG hot uptake at corresponding area.

**Figure 5:**
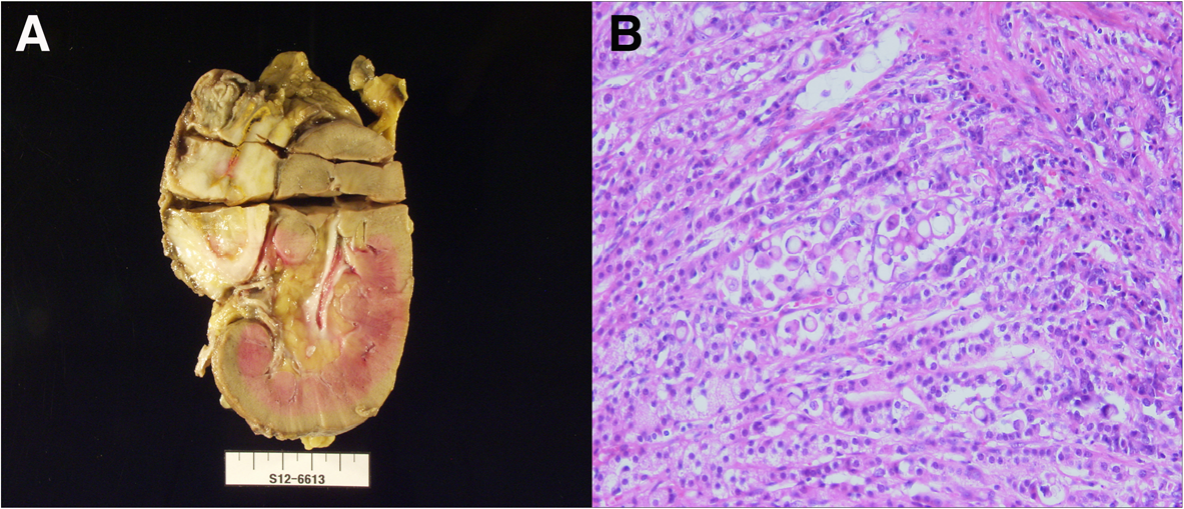
**Photograph and histological tissue specimen of resected tumor from patient 2 shows a 6 × 3–cm adrenal mass without definite direct invasion into the renal parenchyma. (A)** Photograph of the resected tumor. **(B)** Histological evaluation revealed a signet ring cell carcinoma, which had the same histology as the primary lesion (hematoxylin and eosin stain; original magnification, ×400).

A 45-year-old woman underwent distal gastrectomy for AGC at our hospital in July 2006 (pT2bN0M0, stage IB, according to the UICC classification system [6]). A histological examination of the primary gastric cancer lesion revealed a signet ring cell carcinoma that had infiltrated the subserosal layer. There was no lymph node metastasis in any of the 44 retrieved nodes, and the proximal resected margin was 2.0 cm. The patient remained well until 1 year after surgery, when recurrent remnant gastric cancer invading the distal pancreas was detected during a follow-up evaluation. After no other metastasis was identified, a complete, total gastrectomy with distal pancreaticosplenectomy was performed in July 2007. Postoperative adjuvant combination chemotherapy included six cycles of 5-FU and cisplatin. She remained well for 2 years following her second operation until she experienced mild swallowing difficulty. A CT scan and barium swallowing series showed a recurrent mass around the jejunojejunostomy site that compressed the Roux limb and resulted in a luminal obstruction. A third operation was performed in July 2009. *En bloc* resection of the recurrent tumor, including the Roux limb, was carried out, and a repeat esophagojejunostomy with jejunojejunostomy was performed. She refused postoperative adjuvant chemotherapy and remained well for 3 years. However, a 3-cm left adrenal mass found on a CT scan showed intense FDG uptake on PET-CT scans (Figure 4). In this patient, sufficient clinical information was available for surgical resection and other alternative treatments, so surgical resection was planned with the patient’s informed consent. A fourth operation was performed in May 2012, including a left nephroadrenalelctomy, transverse colectomy and resection of the Roux limb because of direct invasion of the adrenal tumor into the renal capsule and adjacent jejunal mesentery and mesocolon (Figure 5A). A histopathological examination revealed a 6 × 3–cm adrenal tumor with metastatic signet ring cell carcinoma, which is the same histologic finding as that of primary and recurrent lesions (Figure 5B). The regional lymph nodes, including jejunal mesentery (0 of 13), transverse mesocolon (0 of 1) and renal parenchyma were free from metastasis or direct tumor invasion. The patient’s postoperative course was uneventful, and she was discharged on postoperative day 10 without any complications. She received second-line chemotherapy with seven cycles of 100 mg/day S-1. During the 12-month follow-up period, no definite evidence of tumor recurrence was found.

## Discussion

Adrenal metastasis most commonly develops from lung cancer, renal cell carcinoma, gastric cancer or esophageal cancer [7,8]. Although some studies have shown less than 10% adrenal metastasis in patients with gastric cancer [9,10], recent autopsy and clinical studies have revealed 16% to 18% adrenal metastasis in patients with gastric cancer [3,4]. There are only a few treatment options available when adrenal metastasis develops, because the prognosis is poor when a local recurrence or distant metastasis develops in a patient with gastric cancer [2]. If a solitary adrenal metastasis develops without any other metastatic foci, surgical resection can be applied to achieve cure. However, a solitary adrenal metastasis is a very rare manifestation in a patient with gastric cancer. In addition, our patients presented with unusual clinical courses.

The role of surgical treatment for adrenal metastases is still evolving, but several authors have advocated resection of adrenal metastases with curative intent in selected patients when no other evidence of metastatic disease exists. Although adrenalectomy for metastatic gastric cancer has not been widely performed, surgical resection has been adopted in many more cases, particularly in cases of lung cancer and renal cell carcinoma [11,12]. In general, adrenalectomy for a metastatic tumors is associated with a poor prognosis: The overall 5-year survival rate is 13% to 29%, and the median survival time is 13 to 28 months [12,13]. However, only surgical resection can cure a solitary adrenal metastasis. Actually, compared with patients who undergo nonsurgical treatment for adrenal metastasis, surgically treated patients have better survival [12,14]. In particular, an adrenalectomy helps to prolong survival in patients with a disease-free interval of more than 6 months and completely resectable metastatic foci. If a solitary metastatic lesion is found within a sufficient period of time from the initial operation, an adrenalectomy will be more effective. Whether induction chemotherapy is necessary depends on tumor size and resectability.

Only three case reports have been published regarding long-term survival following curative resection for solitary adrenal metastasis in gastric cancer [5,15,16]. In the absence of a randomized clinical trial or a large case–control series, any patients who achieve long-term, disease-free survival can be assumed to have derived a clinically significant benefit.

It is not be necessary to perform a preoperative biopsy when solitary adrenal metastasis is highly suspected on the basis of imaging studies and clinical situations. We did not seek preoperative histologic confirmation in patient 2.

Both of our cases were of late onset (6 years following gastrectomy) and involved solitary metastases to the adrenal gland. In patient 2, two additional operations were performed for remnant gastric cancer and a jejunojejunostomy site recurrence. To be exact, the tumor in this patient may not have been a late-onset adrenal metastasis, but a missed cancer lesion. However, she had nearly a 3-year relapse-free period without evidence of a remnant cancer lesion visualized on CT and PET/CT scans. Although our patients had relatively short follow-up periods of 13 and 11 months, respectively, both underwent successful curative resection.

## Conclusions

Both of our cases are valuable to report. The management of adrenal metastases from gastric cancer can pose a dilemma for clinicians. This report shows that the surgical treatment of adrenal metastasis from gastric cancer is an option that may prolong survival in selected patients, such as those with completely resectable lesions or those with late metachronous metastases, as in our patients. The clinical courses outlined herein support the feasibility of selective surgical resection for adrenal metastases in patients with gastric cancer.

## Consent

Written informed consent was obtained from the patient for publication of this case report and any accompanying images. A copy of the written consent is available for review by the Editor-in-Chief of this journal.

## Abbreviations

CT: Computed tomography; PET-CT: Positron emission tomography–computed tomography.

## Competing interests

The authors declare that they have no competing interests.

## Authors’ contributions

DJK wrote the manuscript. JHL provided clinical opinions and the histopathologic images. WK revised the manuscript. All authors read and approved the final manuscript.
